# CMA mediates resistance in breast cancer models

**DOI:** 10.1186/s12935-023-02969-9

**Published:** 2023-07-05

**Authors:** Alessia Lo Dico, C. Martelli, F. Corsi, D. Porro, L. Ottobrini, G. Bertoli

**Affiliations:** 1 Molecular Bioimaging and Physiology (IBFM), CNR, Segrate, Milan, Italy; 2 NBFC, National Biodiversity Future Center, Palermo, Italy; 3 Department of Pathophysiology and Transplantation, University of Milan, Segrate, Milan, Italy; 4 Department of Biomedical and Clinical Sciences, University of Milan, Milan, Italy; 5 Surgery Department, Istituti Clinici Scientifici Maugeri IRCCS, Pavia, Italy

**Keywords:** HER2+ breast cancer, Triple negative breast cancer, Luminal A breast cancer, Luminal B breast cancer, Chaperone-mediated autophagy (CMA), Temozolomide (TMZ), Doxorubicin (Doxo)

## Abstract

**Background:**

Breast cancer (BC) is the most common malignancy in women and the second leading cause of cancer-related death; chemoresistance is still a clinical challenge mainly because of the different molecular features of this kind of tumour. Doxorubicin (Doxo) is widely used despite its adverse effects and the common onset of resistance. Chaperone-Mediated Autophagy (CMA) has been identified as an important mechanism through which chemotherapeutics can exert their cytotoxic effects and, in this context, LAMP-2A, the key player of CMA, can be a useful biomarker.

**Methods:**

A cohort of patients and breast cancer cells have been screened for Doxo effect and CMA activation by analysing the LAMP-2A level. Molecular silencing has been used to clarify CMA role in BC responsiveness to treatments. Low Doxo doses were combined with other drugs (TMZ or PX-478, a HIF-1α inhibitor) to evaluate their cytotoxic ability and their role in modulating CMA.

**Results:**

In this paper, we showed that CMA is an important mechanism mediating the responsiveness of breast cancer cell to different treatments (Doxo and TMZ, as suggested by triple negative cells that are TMZ-resistant and fails to activate CMA). The LAMP-2A expression level was specific for different cell lines and patient-derived tumour subtypes, and was also useful in discriminating patients for their survival rates. Moreover, molecular silencing or pharmacological blockage of HIF-1α activity reverted BC resistance to TMZ. The combination of low-dose Doxo with TMZ or PX-478 showed that the drug associations have synergistic behaviours.

**Conclusion:**

Here, we demonstrated that CMA activity exerts a fundamental role in the responsiveness to different treatments, and LAMP-2A can be proposed as a reliable prognostic biomarker in breast cancer. In this context, HIF-1α, a potential target of CMA, can also be assessed as a valuable therapeutic target in BC in view of identifying new, more efficient and less toxic therapeutic drug combinations. Moreover, the possibility to combine Doxo with other drugs acting on different but coherent molecular targets could help overcome resistance and open the way to a decrease in the dose of the single drugs.

**Supplementary Information:**

The online version contains supplementary material available at 10.1186/s12935-023-02969-9.

## Background

Breast cancer (BC) is the most common malignancy in women and the second leading cause of cancer-related death [1]. Its treatment includes surgery, endocrine therapy, chemotherapy, radiotherapy, and targeted therapy in accordance with its molecular and histological classification [2]. Indeed, BC is a highly heterogeneous disease characterized by different molecular features that affect the response to the diverse therapeutic strategies and eventually the clinical outcome. Three molecular features based on the expression of oestrogen receptor (ER), progesterone receptor (PR), and human epidermal growth factor receptor 2 (HER2) are used as prognostic and predictive biomarkers. St. Gallen Consensus 2011 [3] is, to date, the classification system used to categorize BC based on its molecular subtypes according to the *Luminal A* (ER+ /PR+ /HER2−/lowKi-67); *Luminal B* (ER+ /PR+ /HER2−/+ /high Ki-67); *HER2*+ (ER-/PR-/HER2+) and *Triple-negative breast cancers/TNBCs* (ER−/PR−/HER2−) [4].

Doxorubicin (Doxo) is an anthracycline widely used in BC management, although tumours frequently develop resistance to this drug [5–7]. Besides, Doxo induces several serious adverse effects causing cardiotoxicity and myelosuppression [8]. For this reason, a critical challenge in oncology is the possibility to elucidate the mechanisms involved in the resistance and to identify additional therapies to be used in combination with low-dose Doxo, permitting to reduce its toxicity and maintaining, at the same time, a high treatment efficacy with a reduction in the adverse effects [9, 10]. It has been already outlined the TMZ effect in glioma cells [11], showing the selective engagement of CMA pathway in TMZ-sensitive cells but not in resistant cells. In that model, the unblocking of the CMA activity (for example by using H_2_O_2_ in association with TMZ) restores the sensitivity to TMZ of previous resistant cells. Starting from these considerations, in this paper, Doxo efficacy in BC has been studied concerning Chaperone-Mediated Autophagy (CMA) activation. CMA is a selective kind of autophagy already described to be involved with aggressiveness and resistance [12–14]. To be degraded by CMA, proteins must contain the KFERQ domain that can arise from a simple aminoacidic sequence or post-translational modifications. This motif is recognized by different chaperone proteins, such as heat shock protein 90 (HSP90), STIP1 homology and U-Box containing protein 1/C terminus of HSC70-Interacting Protein (STUB/CHIP), and heat shock protein 70 (HSC70) that, in synergy with the Lysosome Associated Membrane Protein 2A (LAMP-2A) [15], shuttle targeted proteins to the lysosomes for their degradation. One of the KFERQ-containing proteins, whose activity has been reported to be modulated by CMA, is the Hypoxia Inducible Factor (HIF)-1α [16]. Since HIF-1α is a transcription factor involved in different pro-tumoural pathways, the possibility to modulate its activity by acting on CMA could be a strategy to limit its function. For example, it has been described in glioma cells that HIF-1α activity decrease, possibly caused by a CMA-driven pathway, is related to tumour responsiveness to Temozolomide (TMZ). It is an alkylating agent belonging to the second-generation imidazotetrazine pro-drugs with high oral bioavailability that is the gold standard treatment for glioma and melanoma, and it has been also proposed in metastatic BC [17, 18]. Indeed, it has been proposed that HIF-1α pharmacological blockage can revert the resistance to chemotherapeutic treatments [11, 19] in cell cultures. HIF-1α is overexpressed in hypoxic conditions and drives the expression of a pool of genes involved in the adaptation of tumour cells to a hypoxic milieu supporting also the ability of cancer cells to escape treatment toxicity [20]. Moreover, HIF-1α plays a pivotal role in cancer onset, development, progression [21–23] and its association with resistance in BC and other pathologies has been molecularly elucidated [24, 25]. On the other hand, HIF-1α decrease is associated with the reduction of cell proliferation and aggressiveness and a corresponding increased susceptibility to radio and chemo-therapeutic treatment protocols. Besides *HIF-1α* silencing is sufficient to completely lose Multidrug Resistance 1 gene (MDR1) expression (the gene that guides the Multi-Drug Resistance, a critical challenge in BC) and to restore the chemo-sensitivity in tumour cells [26, 27].

Here we aim to study the role of CMA in BC cells in relation to their sensitivity to different treatments, and to assess in a cohort of patients the potential role of CMA activity as a molecular biomarker for BC classification, in view of tailoring a treatment concerning BC classification and molecular features [28, 29]. Furthermore, here we suggest HIF-1α modulation to overcome resistance, as already demonstrated in glioma [11, 19, 21]. Finally, since HIF-1α activation has been related also to Doxo resistance in human osteosarcoma cells [30], we examined if reducing HIF-1α activity [19] would improve Doxo efficacy for BC treatment.

## Materials and methods

### Cell lines and reagents

For in vitro studies, we used three categories of breast cell lines. For Luminal A (LumA) phenotype we used T47D (from ICLC cell biobank, Genova, It), for luminal B (LumB) we used the BT474 (kindly provided by Dr. Daniela Gaglio, IBFM-CNR), for HER2+ phenotype we used SKBR3 (ATCC, Manassas, Virginia, USA), and lastly, for TNBC phenotype we used MDA-MB-231 (ICLC). These cell lines have been selected as representative of the BC subtypes, as reported in [31]. MCF-10 (ATCC) cell line was used as control, being representative of a non-tumorigenic epithelial cell line. T47D cells were cultured in High Glucose DMEM (Gibco, Life Technologies, Carlsbad, California, Stati Uniti); SKBR3 and BT-474 in RPMI (Euroclone, Italy) and MDA-MB-231 and MCF-10 in Advanced DMEM (Euroclone, Italy). All of the media were supplemented with 10% heat-inactivated foetal bovine serum, penicillin and streptomycin (50 IU/mL), and 2 mM glutamine (all Euroclone, Italy). The cells were maintained in a humidified atmosphere of 5% CO_2_ at 37 °C.

### BC human tissue samples for ex vivo studies

We used LumA (n = 10), LumB (n = 5), HER2+ (n = 10) and TNBC (n = 10) human tissues and the corresponding non-tumoural clear margins from surgical resections performed from 2011 to 2013 at the Breast Unit of Istituti Clinici Scientifici Maugeri IRCCS, Pavia, Italy. Samples were referred to the biological collection of the “Bruno Boerci” Oncological Biobank for research applications (Istituti Clinici Scientifici Maugeri IRCCS, Pavia), a biobank certified to ISO 9001:2015 and member of BBMRI.it (the italian node of the BBMRI-ERIC, Biobanking and BioMolecular resources Research Infrastructure—European Research Infrastructure Consortium). Upon informed consent from patients, samples were collected, processed and stored at −80 °C as snap-frozen aliquots immediately after surgery, according to the best practices in biobanking (certification ISO 9001:2015). At the time of collection, HER2 enrichment was assessed through immunohistochemistry (IHC) molecular characterization by the Pathology Service (Istituti Clinici Scientifici Maugeri IRCCS, Pavia), according to the clinical guidelines on BC (ASCO—American Society of Clinical Oncology) together with characterization for oestrogen receptor, progesterone receptors and proliferation marker Ki67. Molecular characterization of the tumour samples has been already reported [28]. The samples were used for the isolation of total RNA in real-time PCR (as described in the methods below).

### Cell viability assay

In vitro treatments were used to evaluate cytotoxicity: 10,000 cells/cm2 were seeded in a complete medium and treated for 24 h (h) with 100 μM Temozolomide (TMZ; Merck Darmstadt, Germany), 0.1–10 µM Doxorubicin (Doxo; Merck) or 25 µM PX-478 dihydrochloride (PX; AchemBlock, CA, USA) and cell viability was evaluated using the Trypan blue exclusion test. The coefficient of drug interaction (CDI) was calculated as follows: CDI = AB/(A × B). AB is the ratio of the combination groups to the control group; A or B is the ratio of the single agent group to the control group. CDI indicates: if < 1 a synergistic effect, if CDI = 1 an additive effect and if CDI > 1 an antagonist effect [32].

### RNA extraction and real-time PCR

Total RNA was isolated using TRIzol reagent (Life Technologies, Carlsbad, California, Stati Uniti) and it was reverse transcribed to cDNA using a High-Capacity cDNA Reverse Transcription Kit (Applied Biosystems, Waltham, Massachusetts, USA) following the manufacturer’s instructions. The real-time PCRs were performed in triplicate for each data point using the Sybr Green technique (Promega, Madison, Wisconsin, USA); the oligonucleotides used are shown in Table 1 (all from Eurofins Genomics, Vimodrone, Milan, Italy). The changes in target mRNA content in relation to the β-actin housekeeping gene were determined using the 2^-ΔΔct Method [33].

**Table 1:** Primer sequences

Gene	Forward	Reverse
*β-ACTIN*	TCAAGATCATTGCTCCTCCTG	CCAGAGGCGTACAGGGATAG
*HIF-1α*	TGATTGCATCTCCATCTCCTAC	GACTCAAAGCGACAGATAACACG
*LAMP-2A*	TGCTGGCTACCATGGGGCTG	GCAGCTGCCTGTGGAGTGAGT
*HSC70*	ATTGATCTTGGCACCACCTA	GGGTGCAGGAGGTATGCCTGTGA
*PHLPP1*	CCTACCTTCTCCAGTGCACT	CCAGCAGTTCCAAGTTTCCT
*BAX*	ATGGACGGGTCCGGGGAG	ATCCAGCCCAACAGCCGC
*BAD*	CCCAGAGTTTGAGCCGAGTG	CCCATCCCTTCGTCGTCCT
*BCL-2*	GATTGTGGCCTTCTTTGAG	CAAACTGAGCAGAGTCTTC
*Glutathione*	ATGCTGTGCAGATGGACTTCAACC	TGGATGTCAAACAGACGAGCGGTA
*SOD-1*	AACTGCAACAGCTGTGGGAATCAC	ACATTGCCCAGGTCTCCC

### siRNA transfection

TNBC cells were transfected with 10 nM of *HIF-1α* silencing RNA (siRNA) (Cat. No. GS3091, Qiagen, Hilden, Germania) and Luminal A, B and HER2+ with 10 nM *LAMP-2A* siRNA (custom-made by Eurofins Genomics, Vimodrone, Milan, Italy) or a scrambled negative control (Product No. 1027280, Qiagen) using the Attractene Transfection Reagent (Cat. No. 301005, Qiagen) as indicated by the manufacturer.

### In silico computational analysis

We applied the computational approach to calculate LAMP-2A distribution in BC patients through The University of ALabama at Birmingham CANcer data analysis Portal (UALCAN) analysis (http://ualcan.path.uab.edu/index.html (accessed on 5 June 2021)) [34, 35]. This software is a user-friendly and interactive web source for the analysis of OMICS data. It allows the analysis of the expression levels of LAMP-2A in BC tumour tissue from The Cancer Genome Atlas (TCGA) database, which includes 1081 BC samples. LAMP-2A proteomic expression profile was also investigated on the base of its distribution across primary tumours (n = 125) or normal tissue (18) and across the major subclasses: normal tissue (n = 18), luminal (n = 64), HER2+ (n = 10) and TNBC (n = 16). The UALCAN-CPTAC (Clinical Proteomic Tumor Analysis Consortium) or UALCAN-CTGA database was used to perform analyses on protein or transcript datasets, respectively.

### Statistical analysis

The in vitro experiments were repeated three times and led to reproducible results. The data are presented as the mean values ± standard deviation (SD) of three independent experiments and were statistically analysed using a t-test or one- or two-way analysis of variance (ANOVA), followed by Dunnett’s or Bonferroni’s multiple comparisons and Prism 4 software (GraphPad Software Inc., San Diego, CA, USA).

## Results

### Doxo effect in BC cells and CMA activity

**Fig. 1:**
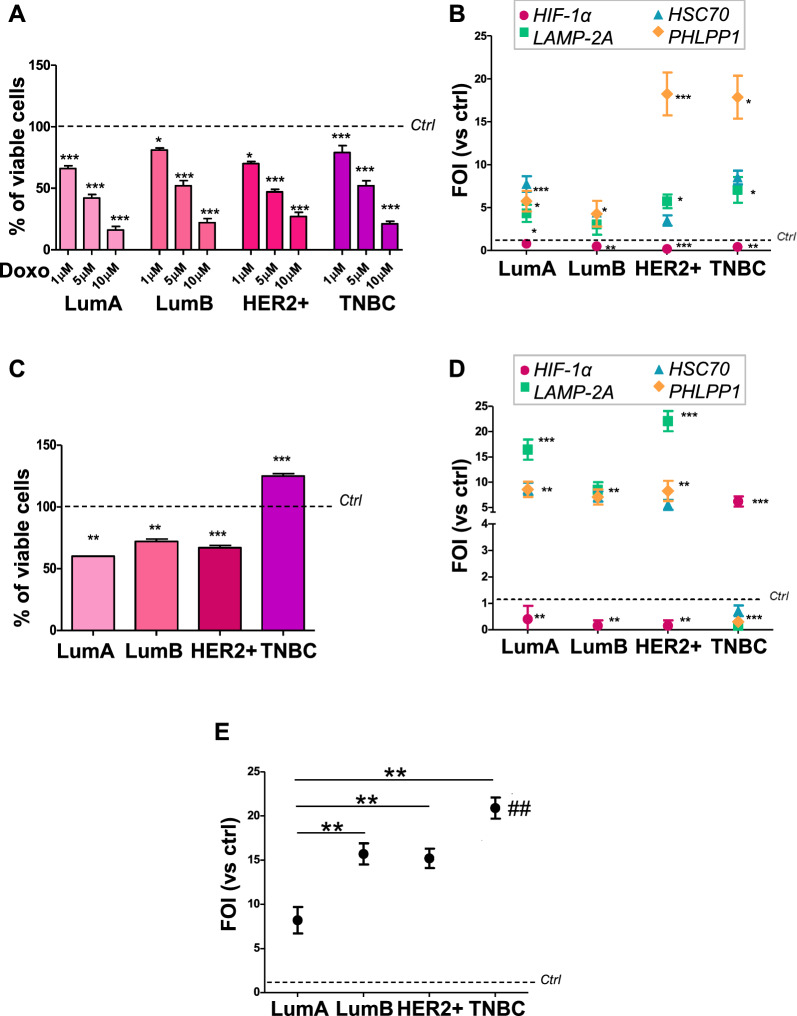
CMA mediated Doxo cytotoxic effect

With the aim of investigating if CMA activity is related to BC responsiveness to standard chemotherapy, we tested the effect of Doxo in a panel of 4 different BC cell lines. All BC cell subtypes were exposed to increasing doses of Doxo (1–10 µM) for 24 h and then cell viability was assessed. In all the cells, we observed a dose–response effect of the treatment (Fig. 1A). To understand if the cytotoxic effect is in relation to CMA activation, we tested the CMA-related genes in all the BC cell lines treated with Doxo 1 µM, as minimal dosage able to affect cell viability. Real time-qPCR (RT-qPCR) revealed a reduction of *HIF-1α* expression in all the cell lines after exposure to Doxo and a concomitant up-regulation of the genes involved in CMA machinery (*LAMP-2A*, *HSC70*, and *PH domain and leucine rich repeat protein phosphatase 1 (PHLPP1)*) (Fig. 1B). The responsiveness of the four BC cell lines to TMZ was studied, showing different responsiveness to TMZ treatment according to their molecular classification. Cell viability revealed that T47D-LumA, BT-474-LumB and SKBR3-HER2+ groups were significantly responsive to TMZ exposure for 24 h. On the other hand, MDA-MB-231-TNBC cells were refractory to treatment, showing a significant increase in cell proliferation (Fig. 1C; Additional file 1: Fig. S1 showed the MTT assay, to sustain viability data). To understand if the toxicity of TMZ on BC-responsive subtypes is dependent on the activation of CMA process, we analysed the panel of CMA-related genes through quantitative reverse transcription PCR (RT-qPCR). LumA, LumB and HER2+ cells showed a similar pattern of gene expression with a significant reduction of *HIF-1α* expression and an up-regulation of CMA-related genes (*LAMP-2A*, *HSC70*, and *PHLPP1*). TNBC showed an opposite expression pattern: the resistance to TMZ is associated with the up-regulation of *HIF-1α* expression and with the reduction of all the genes involved in CMA process (Fig. 1D). To analyse if the different responsiveness was due to molecular differences related to CMA, we measured *LAMP-2A* expression at basal level in all the considered cell lines and compared it with the one in normal-like MCF-10 cells. Figure 1E showed a high and significant increase of *LAMP-2A* in all BC cell lines except for LumA cells.

### LAMP-2A expression was a useful biomarker for breast tumour classification in patients

**Fig. 2:**
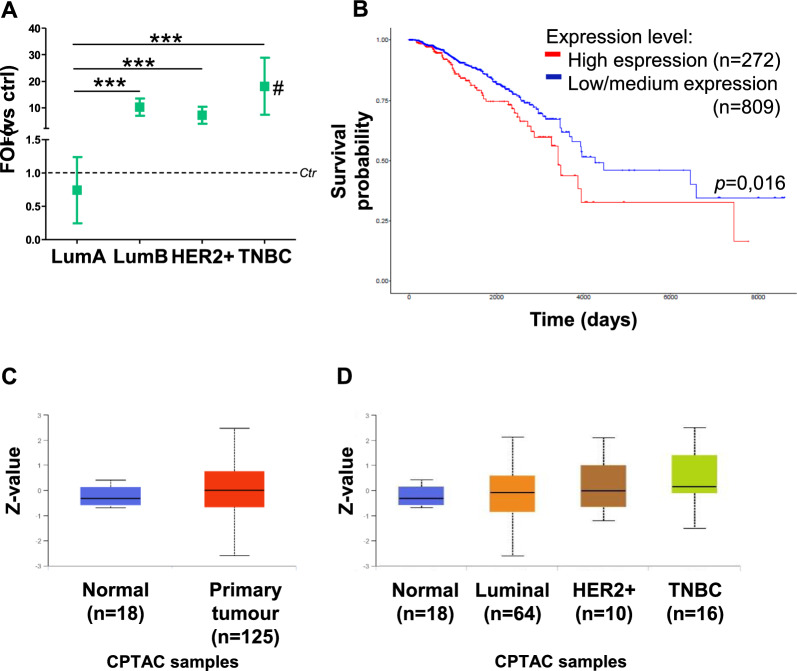
LAMP-2A was a robust biomarker also in breast tissues and in silico

Since in clinical histopathological evaluation LAMP-2A was correlated to the prognosis of different types of cancer including breast cancer [36], we have evaluated the expression level of this gene in patient-derived samples. The RNA analysis of a pool of BC patients showed a different amount of *LAMP-2A* expression concerning the tumour subtype classification (Fig. 2A) and compared to the healthy tissue. In detail, the LumA subgroup exhibited the lowest level of *LAMP-2A* mRNA (Mean Δct value 0,74) compared to LumB (Mean Δct value 10.3) and HER2+ (Mean Δct value 7.2) groups, whereas the highest level of *LAMP-2A* mRNA was detected into TNBC group (Mean Δct value 18).

To assess the role of *LAMP-2A* as a biomarker, its expression was analysed in patient-derived samples and correlated to the survival rate. By examining the overall survival of 1081 breast cancer patients from a public database, The Cancer Genome Atlas (TCGA), we found that *LAMP-2A* level was able to discriminate patient survival by the log-rank test, with a statistically significant *p*-value < 0.0016 (Fig. 2B). In addition, also LAMP-2A protein expression was analysed on CPTAC (Clinical Proteomic Tumor Analysis Consortium) samples, underlying that primary tumours had a significantly higher LAMP-2A protein expression compared to normal tissues (Fig. 2C). LAMP-2A protein level was analysed also in the different BC subtypes, showing a similar trend to that observed in our human samples as regards LAMP-2A transcript. Indeed, patients belonging to the TNBC subgroup had higher and significant LAMP-2A abundance compared to controls (Fig. 2D) confirming results obtained for *LAMP-2A* gene expression. To understand if the treatment with Doxo induced ROS release and its was responsible for CMA activation, we have analysed the level of two key genes of redox-homeostasis, *glutathione* and *superoxide dismutase 1* (*SOD-1*) as representative genes involved in the oxidative defect’s rearrangement, both in breast cancer tissues and cell cultures (Additional file 1: Fig. S2). All tissue samples showed an increase of both genes, data that positively correlated with the high levels of LAMP-2A in the same samples. Also in cell lines, treated with Doxo, concomitantly with the *LAMP-2A* up-regulation we observed an increase in both *Glutathione* and *SOD-1* gene expression. This up-regulation is an index of activation of the detoxification system in response to an increase of ROS levels after treatment. These findings support, even if in an indirect way, the ROS-dependent activation of CMA, and its potential role in tumour cell sensitivity to the treatment similarly to what happens in glioma with TMZ.

### BC responsiveness to TMZ was dependent on LAMP-2A expression

**Fig. 3:**
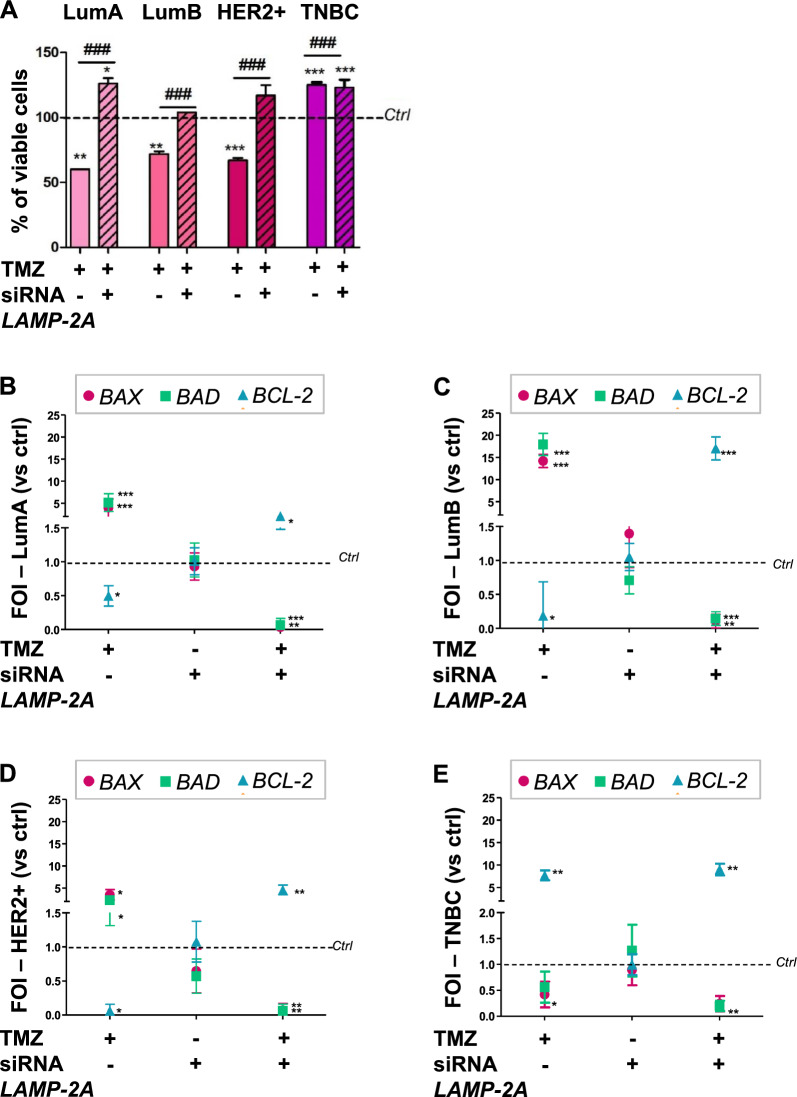
LAMP-2A is necessary to TMZ efficacy in BC cells

To explore the role of CMA in BC, *LAMP-2A* was silenced and cell line responsiveness to TMZ was tested (Additional file 1: Fig. S3 showed the LAMP-2A expression after silencing). In all the cell lines previously sensitive to TMZ, *LAMP-2A* silencing reverts their responsiveness with a significant increase in cell viability (Fig. 3A, ruled columns) while it does not induce any change in responsiveness to TMZ in previously resistant cells. To sustain the cell viability assay, we then evaluated genes involved in apoptosis (*BCL2 associated X -BAX-, BCL2-associated agonist of cell death -BAD- and B-cell lymphoma 2 -BCL-2-*). In all the responsive cell lines, TMZ treatment induced *BAX* and *BAD* expression, whose activity is linked to pro-apoptotic pathway switch-on, and a concomitant reduction of the anti-apoptotic gene *BCL-2* (Fig. 3B–D). In TNBC cells, resistant to TMZ, the drug-induced a reverse expression pattern for *BAX*, *BAD* and *BCL-2* genes, compared to responsive cells. *LAMP-2A* silencing induced a significant change in the expression patterns of these genes determining a resistant-like profile even in previously responsive cells, while no changes occurred in TNBC-resistant cells (Fig. 3E).

### HIF-1α as a potential therapeutic target in TNBC

**Fig. 4:**
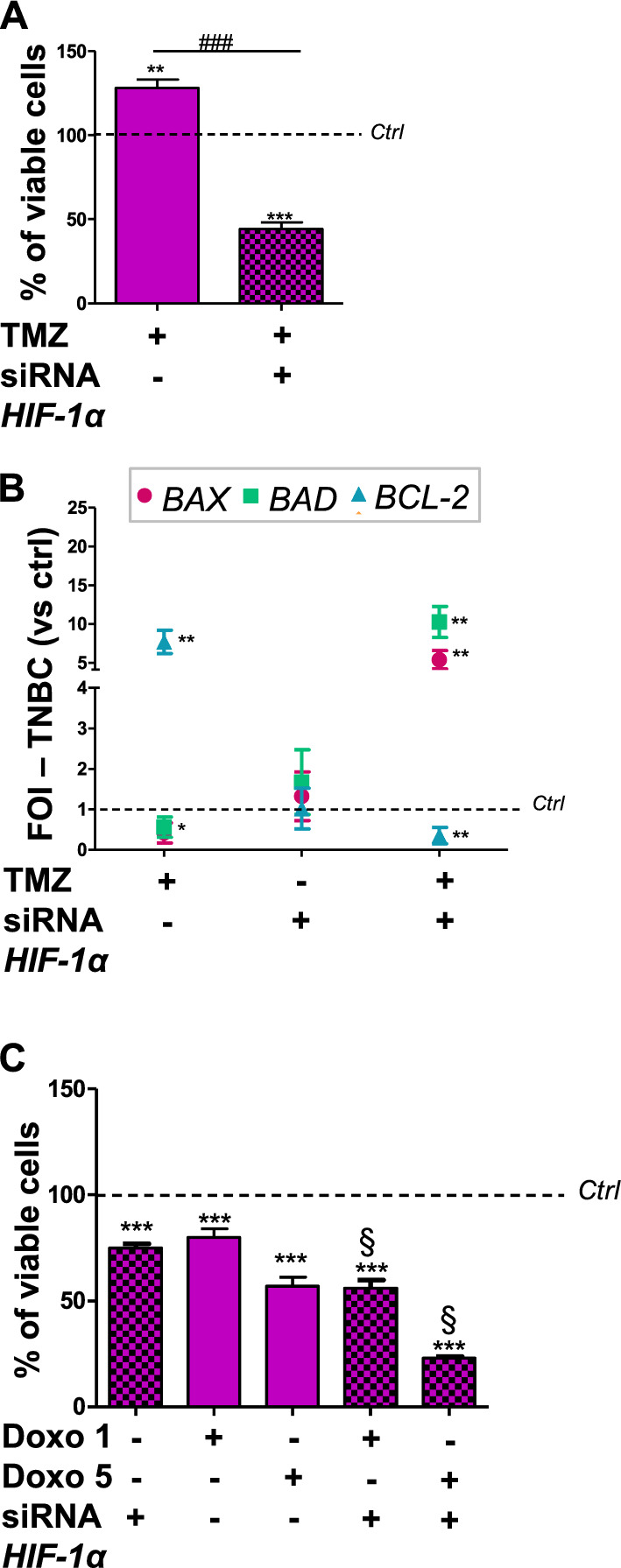
HIF-1α as therapeutic target in BC

Since TNBC cells are resistant to the cytotoxic effect of TMZ and since this drug failed in inducing CMA-related genes, in these cells we silenced *HIF-1α*, a CMA target, with the intent of understanding its role in the responsiveness (Additional file 1: Fig. S4 showed the HIF-1α expression after silencing). We observed that *HIF-1α*-silenced TNBC cells showed a significant reduction of cell viability (Fig. 4A) when treated with TMZ (whereas the silencing alone did not affect the viability compared to scramble-treated cells, data not shown). Also in these cells, we performed an analysis of apoptotic gene expression demonstrating that TMZ treatment alone induced the increase of the anti-apoptotic *BCL-2* gene expression and a reduction of the expression of the pro-apoptotic genes *BAX* and *BAD* (Fig. 4B).

Clinical needs request to reduce the effective dose of Doxo to limit its adverse effects. In this view, in TNBC we combined the lowest doses of Doxo with *HIF-1α* siRNA. Cell viability test suggested that the combined treatment was able to improve Doxo effect in terms of viability reduction, showing a synergistic effect (Fig. 4C).

### Synergistic effect of combined treatments in TNBC

**Fig. 5:**
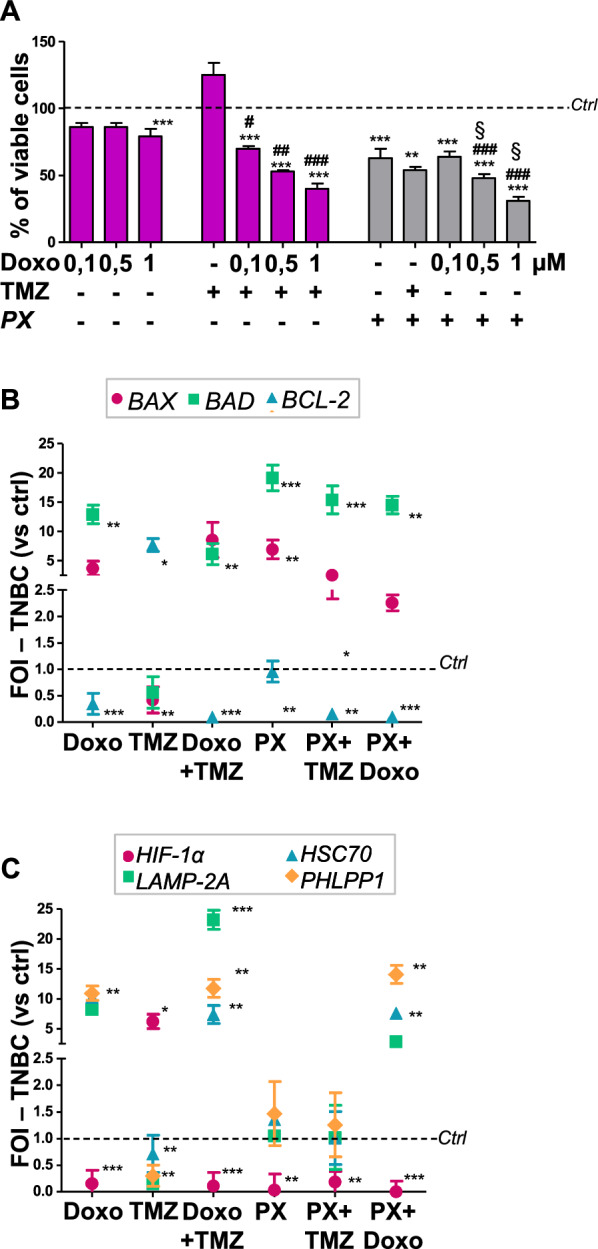
TMZ and PX as adjuvant therapies for Doxo treatment

To better evaluate the contribution of a Doxo-combined treatment to improve therapeutic efficacy, we tested the cytotoxic effect of Doxo in TNBC cells, in combination with TMZ or PX-478 (PX, HIF-1α inhibitor). The combination of Doxo and TMZ produced a significantly higher cytotoxic effect even at the lowest Doxo dose, resulting in a synergistic effect (for 0.1 µM Doxo and TMZ the CDI is 0.65). Similarly, the combination of Doxo with the pharmacological HIF-1α inhibitor PX reduced cell viability, showing again a synergistic effect (CDI for 0.5 µM Doxo and PX is 0.9 whereas with 1 µM Doxo is 0.57) (Fig. 5A). To support the cell viability assay, we also evaluated genes involved in apoptosis (*BAX, BAD* and *BCL-2*) (Fig. 5B). In all treatments that determine a decrease in viability, we observed an induction of pro-apoptotic *BAX* and *BAD* expression, and a concomitant reduction of the anti-apoptotic gene *BCL-2.* Conversely, in TMZ treatment, which did not decrease viability, an opposite trend is reported: high level of *BCL-2* expression and low levels of *BAX* and *BAD* expression. In addition, the decrease in BCL-2 level is more markedly in combined treatment instead of single ones.

The analysis of CMA-related gene expression confirmed this synergy in TNBC cells (Fig. 5C). In fact, even if the Doxo lowest dose (as opposed to TMZ) is able to increase the level of CMA-related genes and decrease *HIF-1α* expression, the combination of Doxo with TMZ showed a further statistically significant increase in the expression of CMA-related genes (*p* < 0.05). As for PX treatment, it is able to down-modulate the expression of *HIF-1α* but does not determine any change in the expression level of *LAMP-2A*, *HSC70* and *PHLPP1.* On the other hand, the combination of PX with Doxo is able to reduce *HIF-1α* expression and induce a positive modulation of CMA-related genes (*p* < 0.05), as opposed to the combination with TMZ.

## Discussion

Breast cancer represents a worldwide problem, being the second leading cause of female cancer death in women (only lung cancer kills more women each year). Even if the mortality rate has been decreasing since 1989, more efforts are still to be made to overcome this statistic [37].

Results reported herein show that CMA is involved in the mechanism through which Doxo can exert its cytotoxicity in breast cancer cells. The critical challenges in breast cancer are overcoming the resistance to standard therapy [4, 38, 39] and reducing the adverse effects of treatments [40, 41]. In this paper, we show that the cytotoxic effect of Doxo goes together with the up-regulation of CMA-related gene profile (*LAMP-2A*, *HSC70* and *PHLPP1*) and with the down-regulation of *HIF-1α* expression. In glioma, it has been already reported that TMZ-responsive cells positively modulate CMA-related genes and abrogate *HIF-1α* expression and activity [11, 19] after treatment with this drug. Since TMZ has been proposed also for the treatment of breast cancer and brain metastasis derived from primary breast cancers [17], we demonstrated that breast cancer cells also could be categorized as TMZ-responsive or -resistant. Moreover, their responsiveness to this drug is in line with their ability to modulate CMA after treatment. In particular, TNBC cells, known to be the most aggressive and resistant subtype, failed in up-regulating CMA-related genes after TMZ treatment, resulting resistant to this drug. These data suggest a key role of CMA in the cell response to TMZ, highlighting once again the importance of this pathway. A role for the CMA-related protein LAMP-2A has been reported in different cancer types, and its expression is related to tumour aggressiveness and poor prognosis [36, 42]. Likewise, here we show by human breast cancer tissues analysis and in in silico evaluations a role for LAMP-2A as theranostic biomarker. In fact, it is able to classify BC subtypes (e.g., TNBC cells showed the highest LAMP-2A expression among the other breast cancer cell lines) and to discriminate patients for their survival. On the other side, modulation of LAMP-2A activity could be proposed as a new therapeutic strategy to overcome resistance. In fact, we have shown that *LAMP-2A* silencing in TMZ-sensitive BC cell subtypes determined the acquisition of a resistant phenotype, indicating that LAMP-2A is necessary to mediate the toxic effect of TMZ, while CMA activation and consequent LAMP-2A activity is fundamental for the exhibition of the toxicity. Even if in our hands it cannot be considered as a direct therapeutic target, these results address the need of new treatments aimed at increasing CMA activity highlighting this pathway involving LAMP-2A as a therapeutic target.

Since previous results obtained in glioblastoma (GBM) cells showed the important role of CMA and suggested HIF-1α as a potential therapeutic target [11, 19], here we also assessed whether HIF-1α abrogation could permit the switch toward responsiveness of TMZ-resistant cells. In TNBC cells, the HIF-1α molecular silencing was sufficient to induce cell responsiveness to TMZ treatment rearranging also the apoptotic gene expression pattern (*BCL-2* down-regulation and *BAX/BAD* induction). Furthermore, the combination of *HIF-1α* silencing together with the lowest dose of Doxo resulted in a synergic cytotoxic effect in TNBC cells. In view of reducing Doxo side effects, which is of great importance in breast cancer treatment, this finding supports the possibility to combine Doxo with other drugs acting on HIF-1α or CMA. For this reason, we combined Doxo with a clinically approved HIF-1α inhibitor, the PX-478, or with TMZ, which had already demonstrated to down-regulate the expression level of this transcription factor in GBM cell lines by CMA induction [11, 19]. Herein, we demonstrated that the combination of both PX-478 or TMZ with the lowest dose of Doxo exerts a synergistic effect on both cell viability and gene expression, confirming the interplay between the mechanisms of action of these two treatments in mediating HIF-1α activity reduction, through CMA activation. By analysing the CMA-related genes, we observed that even if the single treatment with Doxo produced an activated expression pattern of CMA-related genes and a consequent reduction in *HIF-1α* gene expression, the co-treatment with PX-478 does not change the expression pattern, maintaining a responsive-like profile, but shows a more markedly cytotoxic effect. More interesting, the combination between Doxo and TMZ showed that in TNBC cells Doxo at the lowest dose, even if it fails in producing a significant reduction of cell viability, is able to induce an increase in CMA-related gene expression and a decrease in *HIF-1α* gene expression distinctive of a responsive behaviour. By adding TMZ to this drug, there is a synergistic reduction in cell viability and a further increase of CMA-related gene expression (of *LAMP-2A* in particular) confirming the importance of the induction of these genes for the cell responsiveness to the drug.

## Conclusions

In conclusion, in this paper we propose:

- CMA activity as a key mechanism mediating toxic effects of different drugs from one side, and its importance on the ability of several efficacious treatments in inducing this mechanism/pathway, from the other side;
- LAMP-2A as a useful theranostic biomarker in breast cancers, providing clues about BC subtype classification, predicting patient survival, and paving the way for using CMA-inducing drugs as anti-neoplastic treatments;
- HIF-1α activity inhibition as a therapeutic strategy, since its role in driving a wide molecular network supporting cancer cells and involved in BC aggressiveness and in the establishment of resistance.

In this context, we showed that the use of multiple drugs directly or indirectly acting on CMA-related targets could improve cell responsiveness to single treatments and, possibly, could help in reducing side effects by considering combined treatments based on low-dose Doxo. In detail, the association of low-dose Doxo with synergistic drugs acting on different but coherent molecular mechanisms and converging on CMA activation and/or on the reduction of HIF-1α activity, can be pursued to identify new therapeutic options characterized by fewer side effects and less likely to induce resistance.

### Supplementary Information

**Additional file 1: Fig. S1.** MTT assay in TNBC after different treatments. **Fig. S2.** Glutathione and SOD-1 gene expression. **Fig. S3.** LAMP-2A expression after silencing. **Fig. S4.** HIF-1α expression after silencing.

## Data Availability

The data sets used and/or analyzed during the current study are available from the corresponding author on reasonable request. For the in silico computational analysis, we used UALCAN-CPTAC or UALCAN-CTGA dataset from UALCAN (http://ualcan.path.uab.edu/index.html (accessed on 5 June 2021)).

## Abbreviations

ANOVA: Analysis of variance
BC: Breast cancer
BAD: BCL2 Associated Agonist Of Cell Death
BAX: BCL2 Associated X
BCL-2: B-cell lymphoma 2
CDI: Coefficient of drug interaction
CMA: Chaperone-mediated autophagy
CPTAC: Clinical Proteomic Tumor Analysis Consortium
Doxo: Doxorubicin
ER: Oestrogen receptor
GBM: Glioblastoma
HER2: Human epidermal growth factor receptor 2
HER2+: HER2 positive breast cancer
HIF-1α: Hypoxia-inducible factor 1α
HSC70: Heat shock protein 70
HSP90: Heat shock protein 90
IHC: Immunohistochemistry
LAMP-2A: Lysosomal-associated membrane protein 2A
LumA: Luminal A breast cancer
LumB: Luminal B breast cancer
MDR-1: Multidrug resistance 1
PHLPP1: PH domain and leucine rich repeat protein phosphatase 1
PR: Progesterone receptor
PX: PX-478
siRNA: Silencing RNA
RT-qPCR: Quantitative reverse transcription
SD: Standard deviation
SOD-1: Superoxide dismutase 1
STUB/CHIP: STIP1 homology and U-Box containing protein 1/C terminus of HSC70-Interacting Protein
TCGA: The Cancer Genome Atlas
TMZ: Temozolomide
TNBC: Triple-negative breast cancer
UALCAN: The University of ALabama at Birmingham CANcer data analysis Portal
