# Supplementary material for: CMA mediates resistance in breast cancer models
Source: Cancer Cell Int. 2023 Jul 5;23:133. doi: 10.1186/s12935-023-02969-9 (PMC10324152; doi:10.1186/s12935-023-02969-9)
Supplement: Supplementary file 1 — Additional file 1: Fig. S1. MTT assay in TNBC after different treatments. Fig. S2. Glutathione and SOD-1 gene expression. Fig. S3. LAMP-2A expression after silencing. Fig. S4. HIF-1α expression after silencing. [file 12935_2023_2969_MOESM1_ESM.pptx]

## Slide 1
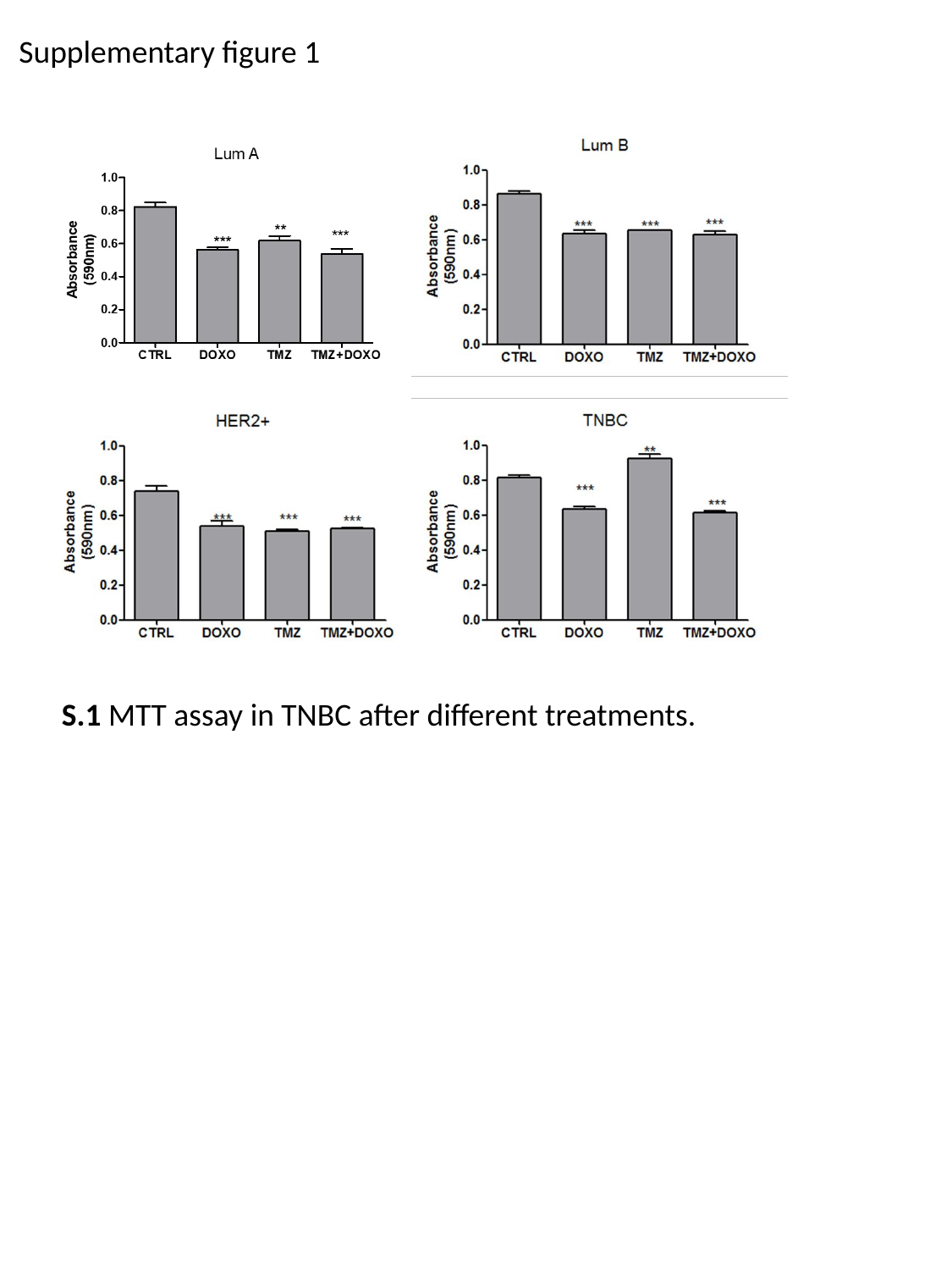

Supplementary figure 1
S.1 MTT assay in TNBC after different treatments.

## Slide 2
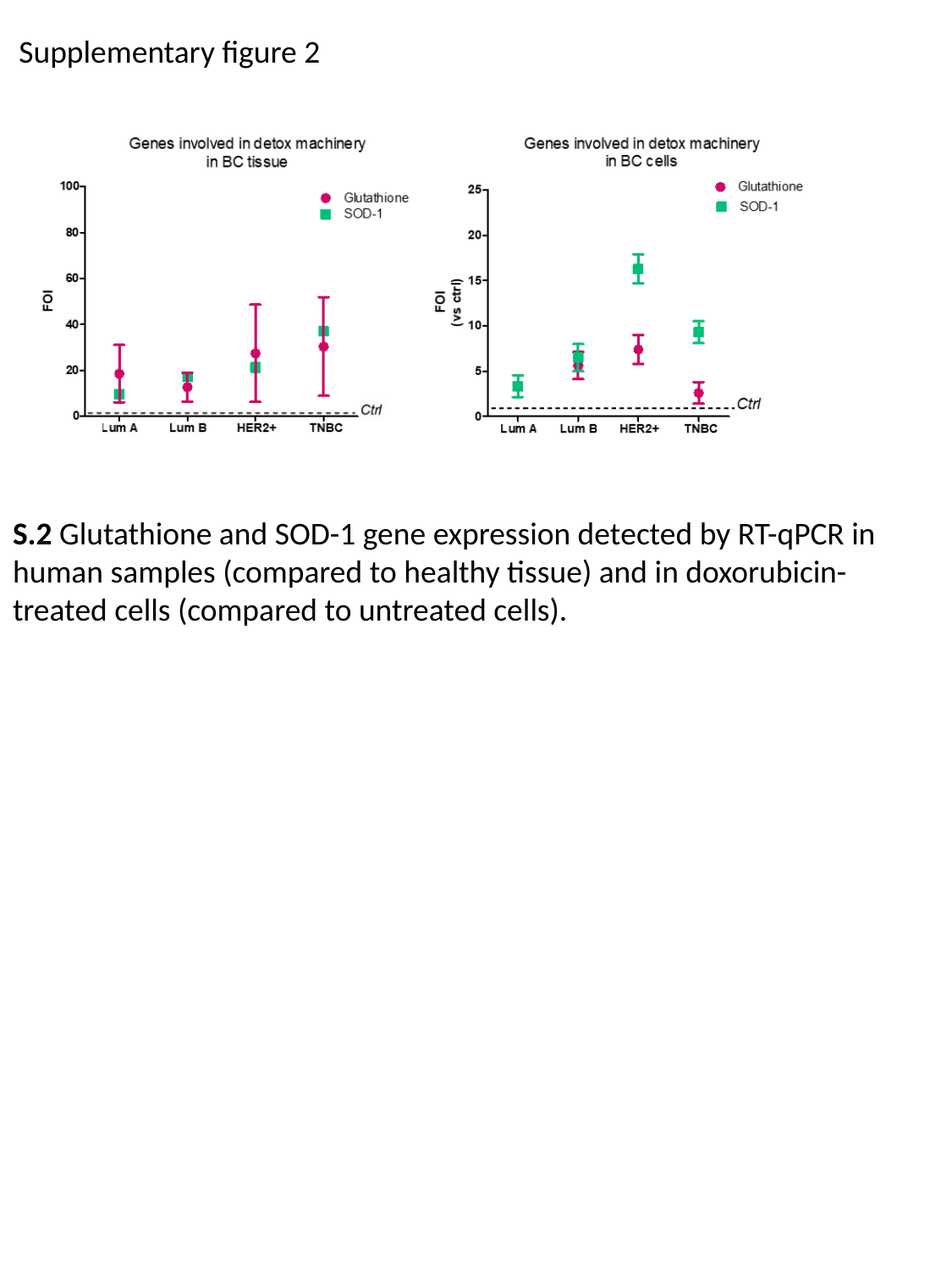

Supplementary figure 2
S.2 Glutathione and SOD-1 gene expression detected by RT-qPCR in human samples (compared to healthy tissue) and in doxorubicin-treated cells (compared to untreated cells).

## Slide 3
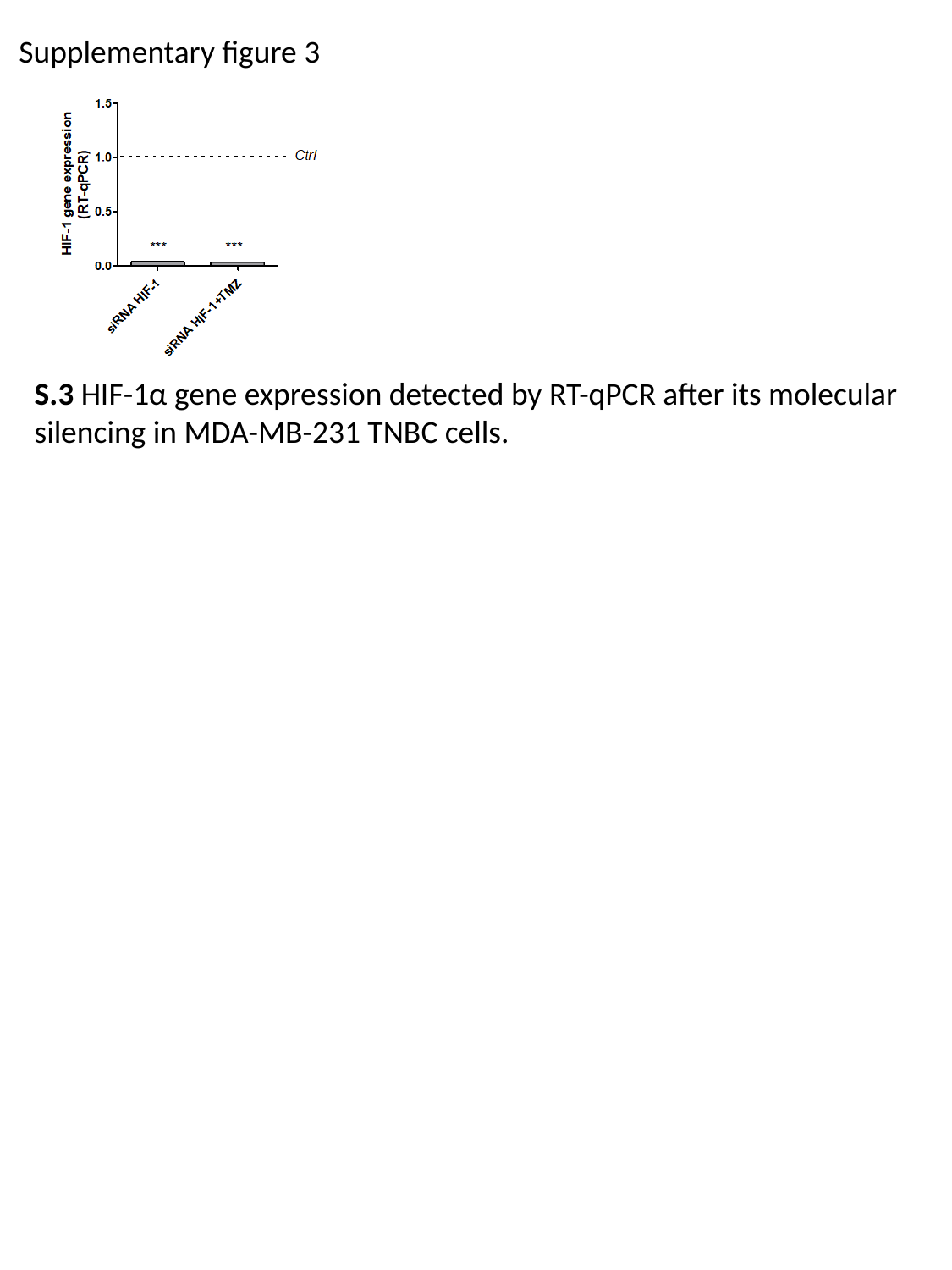

Supplementary figure 3
S.3 HIF-1α gene expression detected by RT-qPCR after its molecular silencing in MDA-MB-231 TNBC cells.

## Slide 4
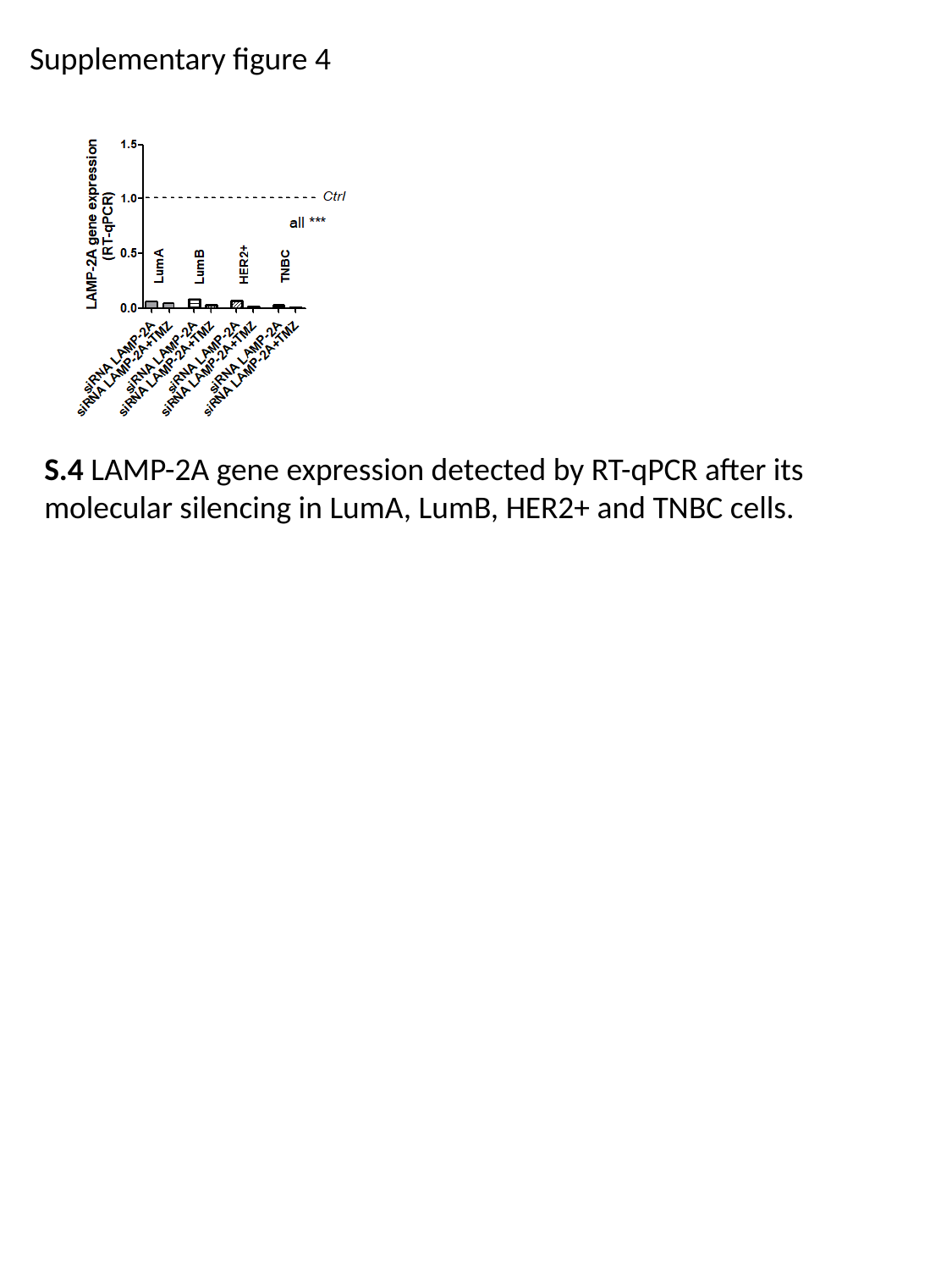

Supplementary figure 4
S.4 LAMP-2A gene expression detected by RT-qPCR after its molecular silencing in LumA, LumB, HER2+ and TNBC cells.
